# Potential disruption of seed dispersal in the absence of a native Kauai thrush

**DOI:** 10.1371/journal.pone.0191992

**Published:** 2018-01-30

**Authors:** Monica Kaushik, Liba Pejchar, Lisa H. Crampton

**Affiliations:** 1 Wildlife Institute of India, Chandrabani, Dehardun, India; 2 Department of Fish, Wildlife and Conservation Biology, Colorado State University, Fort Collins, CO, United States of America; 3 Hawaii Department of Land and Natural Resources and University of Hawaii Manoa, Kaua‘i Forest Bird Recovery Project, Hanapepe, HI, United States of America; Indian Institute of Science, INDIA

## Abstract

Hawaii has experienced a catastrophic decline in frugivorous native birds coupled with the introduction of non-native species. Puaiohi (*Myadestes palmeri*), a critically endangered thrush, is the sole extant native songbird capable of dispersing fleshy fruited plants in the rainforest of Kauai island, Hawaii. As this species has declined to occupy a small proportion of its original range, a suite of largely omnivorous non-native birds have been introduced to this region, including the common and widespread Japanese White-eye (*Zosterops japonicus*). This reshuffling of the bird community could have long-term implications for plant community composition if introduced birds incompletely replace the ecological role of native species. The objective of this study was to evaluate the potential consequences of the local extirpation of Puaiohi for seed dispersal. Specifically, we compared the diet of Puaiohi and Japanese White-eye, vegetation characteristics, and seed rain at sites with and without Puaiohi in the Na Pali-Kona Forest Reserve on the island of Kauai. We found high overlap in the composition of seeds consumed by the two bird species, but differences in the characteristics of seeds consumed; Japanese White-eye appeared more likely to consume smaller seeded species compared with Puaiohi. Sites with Puaiohi received substantially higher seed rain during the study period, despite no significant differences in overall fruit abundance. Our results suggest that non-native birds are unlikely to completely replace the seed dispersal services provided by Puaiohi. If Puaohi continue to be rare and range restricted, we predict a shift in plant community composition through an increase in non-native and small-seeded plants, and possible dispersal failure of other native species. Our findings lend further support to efforts to conserve Puaiohi across its current and former range, and to consider introductions to other suitable areas to ensure the persistence not only of the species and but also its functional role in Hawaii’s montane ecosystems.

## Introduction

The global decline of larger-bodied frugivorous birds has consequences for the abundance and distribution of fleshy-fruited plants [1]. Island ecosystems, which are species-depauperate relative to mainland systems, are particularly susceptible to disrupted seed dispersal mutualisms between plants and animals [2,3]. The Hawaiian Islands are a prime example of an archipelago that has experienced the loss or decline of most larger-bodied avian frugivores; all crow species (*Corvus* spp.) and three of the five endemic Hawaiian thrush species (*Myadestes* spp.) are now extinct in the wild [4–6]. The only thrushes still extant are the Omao (*Myadestes obscurus*) on Hawaii Island and the critically endangered Puaiohi (*Myadestes palmeri*) on Kauai. Both species persist across a fraction of their original range [7], leaving most of Hawaii’s forests bereft of native frugivores. Concurrent to these declines, approximately 58 bird species have been introduced to Hawaii [6], several of which are known seed dispersers [8,9]. Thus, the trajectory of Hawaii’s native plant communities may now be largely dependent on the seed dispersal effectiveness of these introduced birds.

Seed dispersal is an important ecosystem service provided by birds [10,11] that affects the structure [12,13] and diversity of plant communities [14,15], influences the spatial distribution of fruiting plants [16,17], and has important consequences for ecological restoration [18–20]. However, the role of frugivorous birds in seed dispersal differs, even within taxa [21]. The number of seeds dispersed, and the quality of seed dispersal (e.g. movement away from parent plant to suitable microsites) depends on diverse characteristics of the seed disperser, such as gape size [22], fruit handling behavior [23,24], time spent on fruiting tree [25], gut passage time [26] and movement patterns [27]. Many of these traits are strongly correlated with body size [28]. Compared to their small-bodied counterparts, larger bodied frugivores are known to ingest larger seeds in greater numbers, and are also capable of transporting seeds to greater distances [29,30]. Thus, due to differences in body size, bill shape and foraging behavior, introduced birds may disperse different fruits or different numbers of fruits than native birds, which could have important implications for plant communities of a given area.

Understanding the relative contribution of native and introduced birds to seed dispersal is particularly important on the island of Kauai. Due to the synergistic effects of habitat loss and degradation from land use change, invasive species, avian disease and several devastating hurricanes, five of the 13 species of native forest birds known from historic times are extinct [31–33]. Of the two primary frugivores historically present on Kauai, the Kamao or large Kauai thrush (*Myadestes myadestinus*) is believed extinct, whereas the Puaiohi has experienced severe range contraction in recent years [34,35]. The Puaiohi was federally listed as endangered in 1967 [36]. Current estimates indicate a population size of approximately 500 birds with 75% of the total population occupying an area of less than 10 km^2^ on the Alakai Plateau [35,37], a rugged and remote forested plateau above 1000 m asl. Anecdotal observations suggest that this species feeds on a wide variety of native fruit-bearing shrubs and trees [38].

In addition to Puaiohi, five non-native birds have colonized the Alakai Plateau following human-mediated introduction to Kauai. These species, Japanese White-eye (*Zosterops japonicus*), White-rumped Shama (*Copsychus malabaricus*), Melodious Laughing-thrush (*Garrulax canorus*), Japanese Bush Warbler (*Cettia diphone*) and Northern Cardinal (*Cardinalis cardinalis*), are omnivores, and based on their behavior in their native habitat, they have the potential to disperse seeds of fleshy-fruited plants [39,40]. Of these non-native birds, Japanese White-eye is the most widespread and abundant [8]. Although Japanese White-eye have been observed dispersing fruit to various degrees on other islands [8,9,41], their role in facilitating seed dispersal of Kauai’s plants has not yet been evaluated.

Efforts to recover Puaiohi populations have been underway for decades and have included captive breeding and reintroduction as well as habitat improvement through invasive plant and mammal control [42–46]. Although the population is not currently declining, given the critical status of the population and the potential exacerbation of disease risk and habitat loss from climate change, the risk of extinction remains high [37,47,48]. Understanding the ecological consequences of this potential loss for Kauai’s forests is thus both ecologically interesting and conservation-relevant. Here we 1) ask whether species richness and abundance of seed rain differed among sites with and without Puaiohi, and 2) compare the relative abundance and composition of seeds in the diet of Puaiohi and the introduced Japanese White-eye, the most widespread and abundant of the non-native fruit eating birds.

## Methods

### Study area

**Fig 1:**
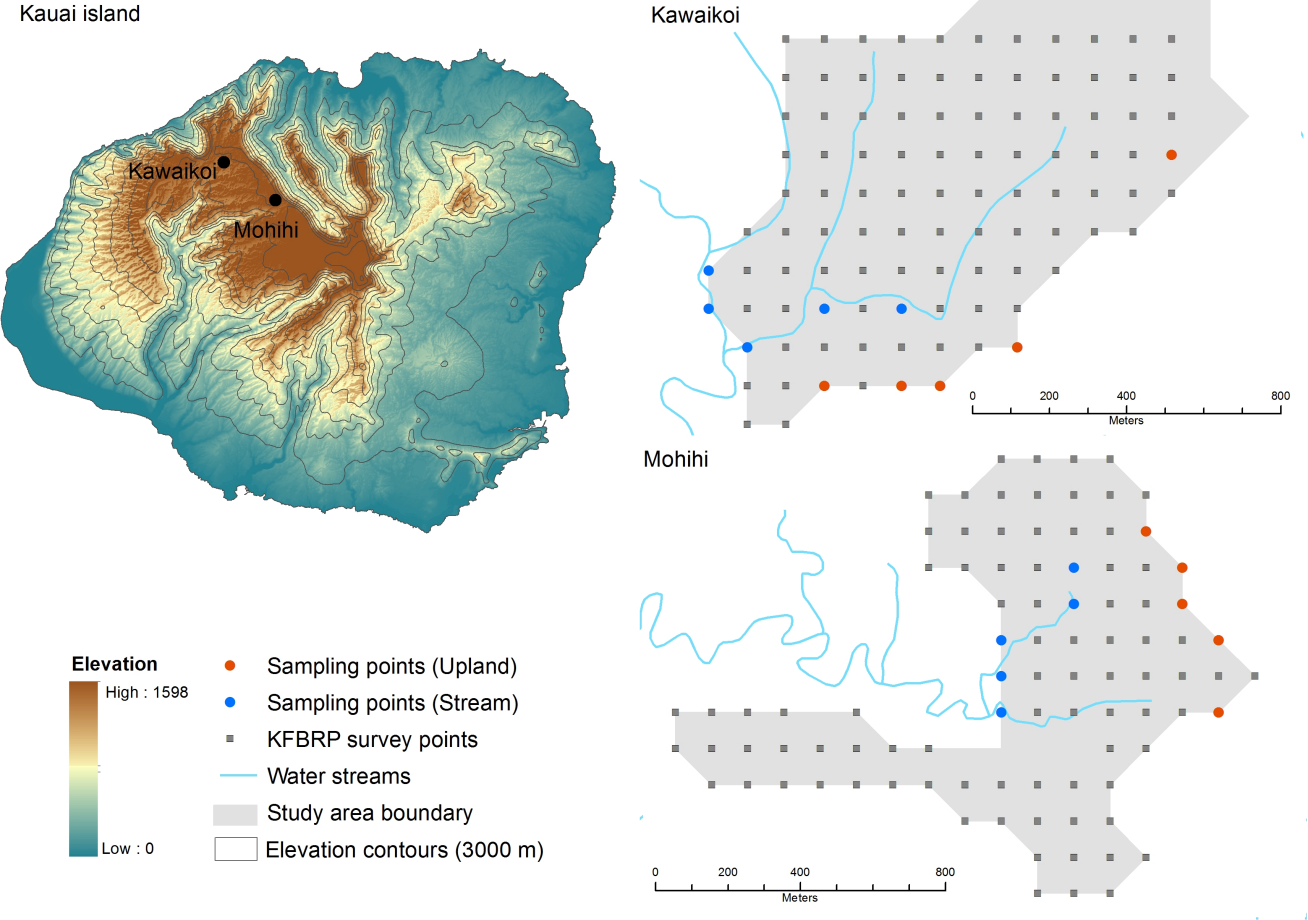
Map of study sites (Kawaikoi and Mohihi) and sampling points on Kauai Island, Hawaii (U.S.). Puaiohi, a native frugivorous thrush, is present in Mohihi but has declined to near extirpation in Kawaikoi. Sampling points are indicated with orange (upland sites) and blue (stream sites) circles. These points are a subset of all Kauai Forest Bird Recovery Project permanent point count stations (grey squares).

We conducted the study in the Na Pali-Kona Forest Reserve on the island of Kauai, Hawaii (U.S.A.) during January–May 2014. Two study sites, Kawaikoi (22°08’21.64” N, 159°35’53.14” W) and Mohihi (22°06’55.82” N, 159°37’32.74” W), were selected within the reserve (Fig 1). Kawaikoi and Mohihi are located at elevations of 1150 m and 1250 m respectively. Average annual rainfall is 2800 mm at Kawaikoi and 3600 mm at Mohihi [49]. Both sites are characterized by wet montane forest dominated by the canopy tree ohia lehua (*Metrosideros polymorpha*), with no differences in ohia lehua canopy cover or tree diameter [50] Other common canopy and sub-canopy trees include ohia ha (*Syzygium sandwicensis*), lapalapa (*Cheirodendron platyphyllum*), olapa (*Cheirodendron trigynum*), alani (*Melicope* spp.), and manono (*Kadua terminalis*). The understory is dominated by woody plants such as ohelo (*Vaccinium calycinum*), kanawao (*Broussaisia arguta*), haha aiakamanu (*Clermontia fauriei*), pilo (*Coprosoma* sp.), pukiawe (*Styphelia tameiameiae*), uki uki (*Dianella sandwicensis*) and ohenaupaka (*Scaevola glabra*). Himalayan ginger (*Hedychium gardnerianum*), thimbleberry (*Rubus parviflorus*) and blackberry (*Rubus argutus*) are introduced invasive plants present at both sites but particularly abundant at Kawaikoi [50]. A third introduced invasive species, strawberry guava (*Psidium cattleianum*), occurs only along a few streams in Kawaikoi. The two sites differ in relative abundance of Puaiohi, the only extant native frugivorous forest bird on Kauai. Until recently, Puaiohi was abundant at both sites but although this species persists in Mohihi, Puaiohi are now extremely rare and possibly extinct in Kawaikoi [38,43]. In contrast, Japanese White-eye, an introduced omnivore, is common and widespread at both sites.

### Study species

The Puaiohi is a medium sized (16·5–18 cm) understory thrush that is endemic to Kauai Island, Hawaii (U.S.). It is largely sedentary with a small home range size (1.2±0.34 ha), and is currently restricted to remote montane forests above 1000m elevation on the Alakai Plateau [42]. This species is frequently observed along streams because it uses cavities in cliff walls for nesting. Anecdotal observations indicate that this species is primarily frugivorous, but also consumes invertebrates during the breeding season, which extends from March- September [51]. The Japanese White-eye is a smaller (10–11.5 cm) bird introduced to the Hawaiian Islands in the 1920s [52]. It is a highly mobile bird with a home ranges that vary in size (0.26±0.24 ha-14.5±9.2 ha) depending upon resources availability [53,54]. It forages in a variety of habitats (closed forest, open woodland, secondary growth, and cultivated areas), and has a generalist diet that includes insects, fruits and pollen [55].

### Sampling design

All sampling points were located in or adjacent to current or previously occupied Puaiohi territories in Kawaikoi and Mohihi. These points were selected from occupancy survey locations previously established by the Kauai Forest Bird Recovery Project (KFBRP) at both study sites [50] (Fig 1). Because Puaiohi are so rare, the best index of Puaiohi relative abundance is occupancy, which was surveyed on both Kawaikoi and Mohihi in 2011. Probability of occupancy was 0.19 (95% CI: 0.14–0.25) in Kawaikoi and 0.75 (95% CI: 0.62–0.88) in Mohihi [37]. In 2011, Kawaikoi held 1–2 territories of Puaiohi; by 2014, KFBRP was unable to detect any breeding Puaiohi on Kawaikoi stream. Conversely, on a similar 3km stretch of Mohihi KFBRP found nine territories in 2011, and by 2013 had found a total of 12 territories, with an additional five territories on side streams (KFBRP unpubl. data).

At each study site five points were located along streams, where Puaiohi typically nest [38,56], and five points were located in upland areas, which are potential Puaiohi foraging areas, for a total of 10 points at each study site. Introduced frugivorous birds nest and forage in both stream and upland areas at both sites (L. Crampton and R. Hammond personal observation). Sampling points were established at least 100 m apart to ensure independence [7,42]. Data on tree and fruit density and seed rain were collected from January-May 2014, which is the peak breeding season for Puaiohi and most other native and introduced Kauai forest birds [57–60].

### Tree and fruit density

We recorded the number of fruiting trees of each species and estimated fruit density on each individual tree within a 3-m radius plot from the center of each sampling point. Fruit density was estimated using the rank-scale method [61]. Using the same methods, we also quantified neighborhood fruiting plant and fruit density within a 10-m radius plot concentric to the 3-m plot. Fruit density for both 3-m and 10-m radius plots were quantified during January-February 2014. From March-May 2014 fruit density was quantified monthly only for the 3-m radius plot. To reduce observer bias in fruit quantification, all measurements were done by MK.

### Bird density

We estimated densities of introduced birds at each study site using variable radius point transect data collected by the Kauai Forest Bird Recovery Project from March-May 2012. Birds were observed on multiple occasions (n = 3) at 29 point count locations in Mohihi and 33 locations in Kawaikoi (Fig 1). Data collected included the radial distance from the observer to each bird, method of detection (visual or aural), the total number of individuals of each species, and other factors such as weather (wind, percent cloud cover and rain) that could have affected detection probability. All point transects were conducted between 0650 and 1230h. Since densities estimated from raw counts may be biased due to differences in detection probability, we accounted for detection bias using program DISTANCE version 6.2 [62]. Because Puaiohi are so rare and their distribution centered around meandering stream channels, traditional methods to measure density are not applicable. Thus we instead relied on previously reported occupancy values for each site, as described above [37].

### Seed rain

We installed four seed traps at each sampling point (n = 40 seed traps per site). Seed traps consisted of a plastic garden pot (35.6cm x 30.5cm) on top of which a cotton cloth was draped to form a collecting bag and fastened using synthetic rope. The weave of the cloth was fine enough to capture the smallest seeds (width ~1mm) while allowing water to flow through. Extra holes were drilled at the base of the pots to prevent the seed traps from becoming flooded during excessive rain. To exclude rats, the seed traps were covered with 1.3 x 1.3 cm metal mesh fastened to the pot with twine. This mesh size was larger than any bird-dispersed seed in the study area. Most seed traps (n = 68) were tied at least 1m off the ground and away from adjacent trees to avoid disturbance by feral pigs. However, to capture seed rain under short-statured vegetation, some traps (n = 12) were set on the ground under fruiting shrubs and fastened with camp stakes to prevent disturbance by pigs. Traps were installed non-randomly under the fruiting trees closest to the sampling points to maximize the probability of capturing bird-dispersed seeds, and were purposely placed under a variety of fruiting plant species around a sampling point [63]. The fruiting plant species above each seed trap was recorded, and all seeds belonging to that species (e.g., seeds that could have fallen from these plants into the trap without being bird-dispersed) were excluded from data analysis. Only seeds without pulp, an indication of bird dispersal, were included in the analysis. Seeds with pulp removed by rats were also excluded. These are easy to identify because they are scarified by rat teeth. The total area sampled by the four traps at each point was 0.40 m^2^; hence the total area sampled in each site was 16.0m^2^.

Traps were checked monthly throughout the field season and the entire cloth containing the seed rain was placed in a clean zip-lock bag. The contents of each bag were sorted beneath a compound microscope and all seeds were separated from other plant debris. Seeds were counted and identified to the lowest possible taxonomic level with the assistance of a previously-assembled seed library. All damaged seeds were also identified and counted to estimate differences in seed predation among sites.

### Bird diet

Collecting a sufficient sample size of fecal samples from Puaiohi and Japanese White-eye during our field season was not practical. Puaiohi occur in low densities so even an intensive mist netting effort, which was beyond the scope of our project, would have led to very few captures. Japanese White-eye forage largely in the canopy, which also makes capture difficult in our remote and densely vegetated sites. However, fecal samples of Puaiohi and Japanese White-eye had been collected opportunistically at our study sites and adjacent areas with similar plant and bird communities by Kauai Forest Bird Recovery Project’s biologists from 2006 to 2014. We used these samples to estimate the degree of dietary overlap between these two species. Puaiohi fecal samples (n = 88) were collected from 2006 to 2012 and Japanese White-eye fecal samples (n = 42) were collected from 2012–2014. Fecal samples for both species were collected in the months October-November and January-June. We counted and identified the seeds in each fecal sample to the lowest possible taxonomic level (genus or species) under a compound microscope using a previously assembled seed library. Finally, the length and width of five randomly selected individual seeds of each species was measured to the nearest 0.5mm using calipers.

### Data analysis

To assess whether site (Kawaikoi, Mohihi) and trap location (upland, stream) influenced seed dispersal (richness and rate of seeds dispersed), we combined seed rain data from all four traps at each sampling point (n = 20 points). Only seeds without pulp, an indication of bird dispersal, were included in the analysis. Seeds from plant species immediately above the traps and with pulp removed by rats were excluded. To calculate the rate of seeds dispersed, and ensure these values were comparable across the two sites, the total number of seeds collected per point was divided by the total number of trap days. The final value included in the analysis was thus seeds per m^2^ per day, which we term “rate of seed dispersal”. Similarly point-wise seed species richness was calculated by pooling the number of seed species found in all four traps at each point over the entire study period. We compared differences in seed dispersal rates and species richness across the two sites using a non-parametric Mann–Whitney test. To investigate the factors responsible for differences in these seed dispersal metrics among the two sites, we used general linear models with Gaussian (rate of seed dispersal) and Poisson (seed species richness) error distribution. We fit eight models that encompassed our *a priori* predictions of the several factors most likely to influence seed rain: (1) null (intercept only); (2) trap location (stream vs upland); (3) site effect (e.g. presence/absence of Puaiohi); (4) interaction of trap location and site effect (Site × Trap location; where × represent interaction term); (5) within plot fruit density; (6) fruit density of non-native *H*. *gardenerium;* (7) density of native fruits; and (8) a full model containing all of the above variables. The densities of native and non-native seeds dispersed were also modeled separately with the same set of explanatory variables. Akaike information criterion for small sample sizes (AICc) was used for model selection since the ratio of sample size (n) and number of parameters (K) was small (<40;[64]). All statistical analyses were performed using program the R version 3.0.2 [65] with packages ggplot2 [66] and MuMIn [67].

We investigated diet similarity between the Puaiohi and Japanese White-eye using Pianka’s index:

$$\overset{n}{\underset{i=1}{\sum }}pij.pik/\sqrt{\overset{n}{\underset{i=0}{\sum }}\left(pij^{2}\right).{\left(pik\right)}^{2}}$$

Where j is species 1, k is species 2, and pij is the frequency of occurrence of prey item i in the diet of species j [68]. Pianka’s index ranges from 0 to 1, where 0 indicate no overlap and 1 indicate complete overlap. We used χ^2^ analyses to compare the frequency of occurrence and relative abundance of different seed species in Puaiohi and Japanese White-eye fecal samples.

## Results

### Tree, fruit and bird density

Sites with and without Puaiohi did not differ in fruiting plant species richness (Kawaikoi = 3.0_Mean_± 0.72_SE_, Mohihi = 1.11_Mean_± 0.13_SE_; W = 32, p = 0.18). Although Mohihi had higher density of fruiting plants (W = 23, p = 0.02) and native plant density (W = 17, p = 0.005), neither fruit density within 3m of sampling points (Kawaikoi = 103.6_Mean_± 51.3_SE_, Mohihi = 53.2_Mean_± 20.2_SE_; W = 61, p = 0.42) nor within 10m of points (Kawaikoi = 250.4_Mean_± 8.13_SE_, Mohihi = 224.1_Mean_± 6.05_SE_; W = 45, p = 0.73) differed between the two sites. Fruit densities of particular plant species were similar between the two sites with the exception of two plant species. *H*. *gardnerianum*, a non-native invasive shrub, occurred in higher densities at Kawaikoi (Kawaikoi = 4.3_Mean_±1.54_SE_, Mohihi = 0_Mean_±0_SE_; W = 85, p = 0.001), and *B*. *arguta*, a native shrub, at Mohihi (0_Mean_+0_SE,_ Mohihi = 25_Mean_+11.5_SE_; W = 30, p = 0.00) (S1 Table).

Density of all non-native birds (individuals/ ha (%CV)) was similar across the two study sites [Kawaikoi = 9.96 (14.4), Mohihi = 9.87 (15.0); t = 0.08, df = 6, p = 1.08]. Density of Japanese-White-eye between sites was also similar (Kawaikoi = 7.86± 0.10; Mohihi = 8.71±0.12; t = 1.30, df = 6, p = 0.65), and consistent with values reported by other investigators in the same study region [69].

### Seed rain

We found 322 bird-dispersed seeds in the seed traps belonging to nine plant species across both sites during the four-month study period (Table 1). Of these nine plant species, six were native and three were introduced. Seeds belonging to six plant species (four native; two introduced) were collected from Kawaikoi, whereas seeds of six species (five native; one introduced) were collected from Mohihi (Table 1). Seeds were primarily from the native species *B*. *arguta* (67%), *C*. *trigynum* (10%), *V*. *calycinum* (8%) and *C*. *platyphyllum* (8%). Seeds of *R*. *parviflorus*, an introduced shrub, were collected only from Mohihi, and contributed 6% towards the total seed rain. Seeds from exotic plants *H*. *gardnerianum* and *R*. *argutus*, and native plants *C*. *fauriei* and *P*. *sandwicensis*, each contributed <1% to the total seed rain.

**Table 1:** The number of bird-dispersed seeds in seed traps at a study site with only introduced frugivorous birds (Kawaikoi) and a site with both native and introduced frugivores (Mohihi). The total number of seeds at each site, the rate of seed dispersal, and the proportion of points with traps that contained seeds are provided.

Plant Species	Number of bird-dispersed seeds in traps	
	Kawaikoi	Mohihi
**Native**		
*V*. *calycinum*	0	23
*C*. *platyphyllum*	2	31
*C*. *trigynum*	3	22
*B*. *arguta*	3	212
*C*. *fauriei*	1	0
*Perrottetia sandwicensis*	0	2
**Introduced**		
*R*. *parviflorus*	0	19
*H*. *gardnerianum*	1	0
*R*. *argutus*	3	0
—————————————————— Summary Statistics	—————————	—————————
Total # of seeds in traps	13	309
Rate of seed dispersal (seeds/day/m^2^)	1.71	40.05
Percentage of traps with seeds	25	33
Percentage of sampling points with seeds	13	80

**Fig 2:**
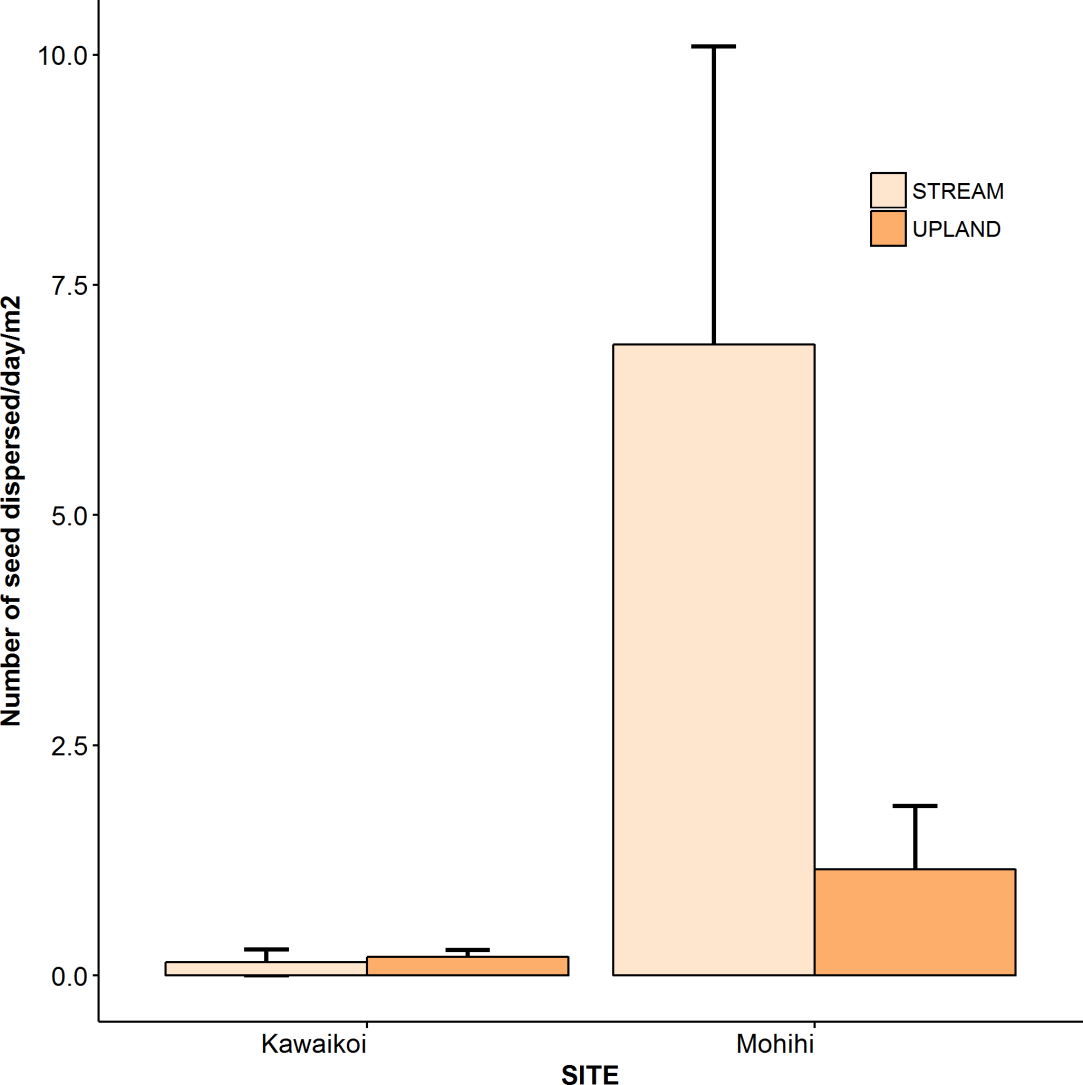
Mean (±SE) seed dispersal rate (seeds/day/m^2^) of bird-dispersed seeds in seed traps at stream and upland sampling points in Kawaikoi (introduced frugivorous birds only) and Mohihi (native and introduced frugivorous birds) on the island of Kauai.

Of the candidate models explaining observed differences in rates of seed dispersal, the model that included site (Kawaikoi or Mohihi) and an interaction of site with trap location (stream or upland) best explained the data (Table 2; Fig 2). The top model explained 38% of the total variation in the data. The rate of seed dispersal was 31 times higher at Mohihi than Kawaikoi (Mohihi: 0.62 seeds/day/m^2^; Kawaikoi: 0.01 seeds/day/m^2^; Table 3). Moreover, the rate of dispersal was 19 times higher in the seed traps located near streams than in the traps in the upland areas at Mohihi (Table 3). In contrast, at Kawaikoi the rate of seed dispersal was somewhat lower (0.26 times) along streams compared to upland areas. Mohihi had a 61 times higher rate of seed dispersal at stream trap locations compared with Kawaikoi. Rates of seed dispersal at upland trap locations did not vary substantially between sites; the rate was somewhat higher (1.21 times) at Kawaikoi.

**Table 2:** A priori hypothesized models built to explain the rate and richness of seeds dispersed in seed traps at study sites in the Alakai Swamp, Kauai. Models are ranked by scores of Akaike’s information criterion adjusted for small sample size (AICc).

Rate of seed dispersal (seeds/m^2^/day)				Seed species richness			
Model^a^	AICc	ΔAICc	*Wi*	Model	AICc	ΔAICc	*wi*
Site	35	0	0.47	Site	34.6	0	0.35
Site ×Trap location	35.6	0.6	0.35	NULL	35.4	0.84	0.23
NULL	39	4	0.06	Trap location	36.8	2.19	0.12
*H*. *gardenerium* fruit density	39.4	4.4	0.05	Site ×Trap location	36.9	2.32	0.11
Trap location	40.4	5.4	0.03	*H*. *gardenerium* fruit density	37	2.43	0.10
Native Fruit density	41.5	6.5	0.02	Native Fruit density	38.1	3.54	0.06
Total fruit density	42.5	7.5	0.01	Total fruit density	40.1	5.57	0.02
Full Model	44.1	9.1	0.01	Full Model	45.6	10.99	0.00

^a^ Models investigate the effects of sampling sites Kawaikoi (introduced frugivores only) and Mohihi (native and introduced frugivores), trap locations (stream vs upland), native and non-native fruit density in 2014. Columns include the covariates used in the model including intercept, AICc score, distance from the lowest AICc (ΔAICc) and Akaike’s model weight (*wi*). Site refers to Mohihi (Puaiohi present) or Kawaikoi (Puiaohi rare or absent), and Trap Location refers to traps at stream or upland locations.

**Table 3:** Summary of linear model results showing model averaged coefficient estimates for the rate of seed dispersal.

Variables	β-estimate	Adjusted SE	t-value
**Intercept**	0.12	0.19	0.59
**Site (Mohihi)**	0.78	0.34	2.3
**Trap location (Upland)**	0.11	0.32	0.35
**Site (Mohihi) × Trap Location (Upland)**	-0.80	0.45	1.81

Although, seed species richness in traps differed marginally between Mohihi and Kawaikoi (W = 25, p = 0.05), variation in seed richness was not strongly explained by any of the covariates used for model building as the null model was within 2 ΔAICc of the top model (Table 2). Seed traps located near streams and upland points also did not differ in seed species richness for either site (Mohihi: stream = 2.2±1.30, upland = 1±0.71; W = 19.5, p = 0.17; Kawaikoi: stream = 0.8±1.79, upland = 0.8±; 0.84; W = 9, p = 0.48).

In addition to bird-dispersed seeds, we also found a total of 667 seeds that showed evidence of damage by rats (e.g. seeds fragmented) in the seed traps. The majority (97%) of the damaged seeds were from *C*. *trigynum*, followed by 2% from *R*. *argutus*, and 1% from *C*. *platyphyllum*. More rat-damaged seeds were found in traps within Kawaikoi (42.8_Mean_ ± 24.17 _SE_) than Mohihi (6.1_Mean_ ± 5.47 _SE_), and in traps located along streams (39.5_Mean_ ± 23.5 _SE_) than upland areas (9.4_Mean_ ± 9.17 _SE_). However, these differences were not statistically significant (W = 66, p = 0.16).

### Bird diet

**Fig 3:**
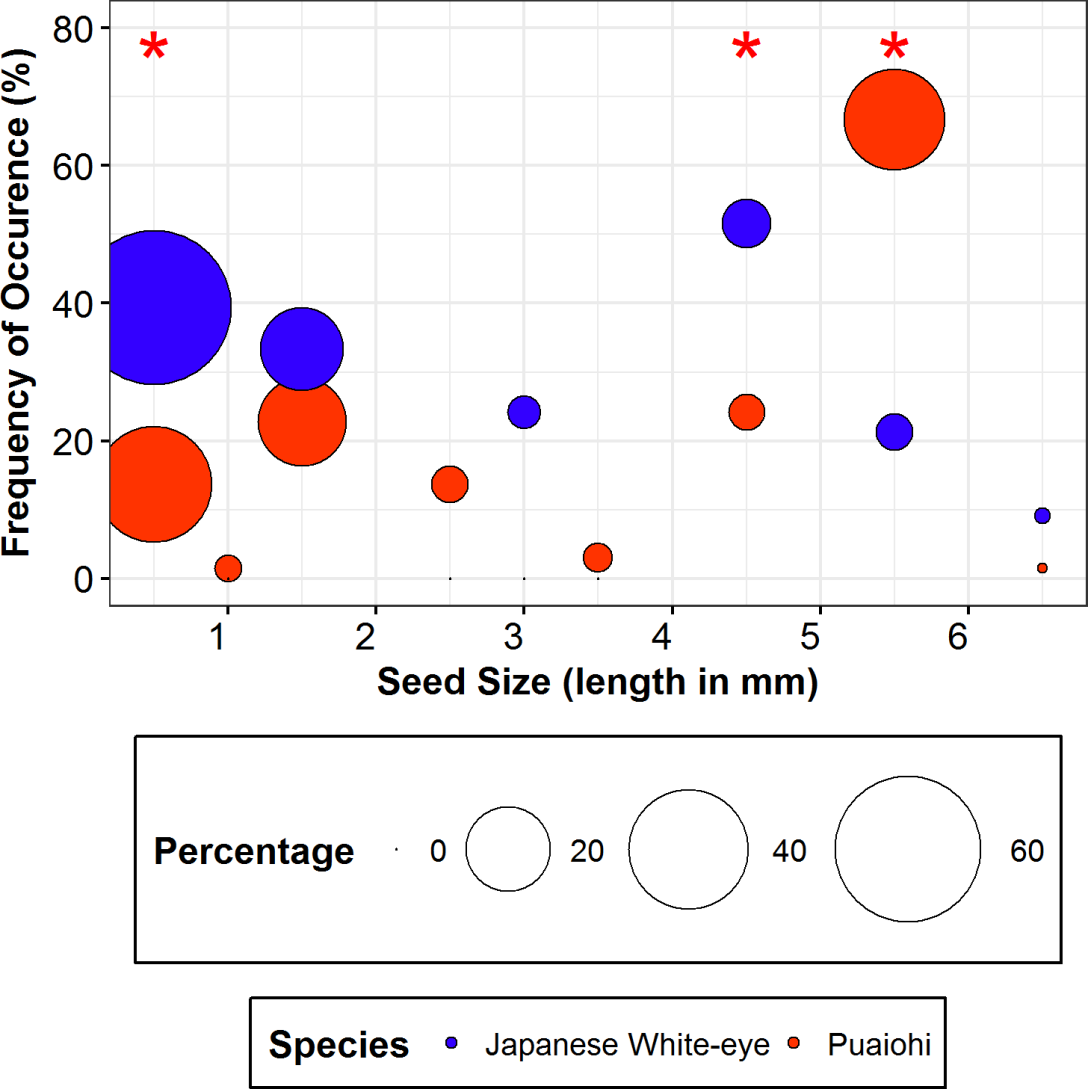
Frequency of occurrence of seeds in Puaiohi and Japanese White-eye fecal samples in different size categories. The size of each bubble represents the relative abundance of seeds in a given size category for each species. Larger bubbles indicate higher relative abundance. Statistically different frequencies within seed size categories for Puaiohi and Japanese White-eye fecal samples are indicated with an asterisk *.

Seeds were found in the majority of both Puaiohi and Japanese White-eye fecal samples (Japanese White-eye = 79%, n = 42; Puaiohi = 75%, n = 88). Japanese White-eye dispersed seeds included 10 plant species of which three are introduced, whereas Puaiohi dispersed seeds came from 11 native species and no introduced species (Table 4). Sizes of seeds in fecal samples varied from the relatively-large seeded *Coprosma kauensis* (seed length = 6.5 mm, width = 5mm) to small-seeded *B*. *arguta* (seed length = 0.5 mm, width = 0.25mm; Table 4). Pianka’s index indicated a high degree of overlap between Puaiohi and Japanese White-eye in terms of seed species and the average number of seeds in fecal samples (S2 Table). However, the two bird species differed considerably in the relative proportion (χ^2^ = 43.85, d.f. = 15, P = 0.0001) and frequency of occurrence (χ^2^ = 119.28, d.f. = 15, P <0.0001) of seeds from various plant species in their diet. The majority (84.4%) of the seeds in the Japanese White-eye fecal samples consisted of two small-seeded native shrub species (*B*. *arguta* and *V*. *calycinum*), with the remaining fraction distributed among larger-seeded native (9.7%) and introduced plants (5.9%; Table 4). In contrast, 59.6% of the seeds in Puaiohi fecal samples consisted of small-seeded species (*B*. *arguta and V*. *calycinum*), while >40% of the seeds were from larger-seeded native shrubs (*R*. *hawaiensis*, *S*. *tameiameiae*) and subcanopy trees (*C*. *trigynum*, *C*. *platyphyllum)*. These differences in the frequency of seeds belonging to different size categories in bird fecal samples were significant (χ^2^ = 93.46, d.f. = 8, P<0.0001; Fig 3).

**Table 4:** The number (N), relative abundance (%), and relative frequency (%) of seed species in the fecal samples of Japanese White-eye (42) and Puaiohi (88). Only fecal samples that contained seeds are included.

Plant Species			Japanese White-eye			Puaiohi	
	
	Seed size^b^ (mm)	N	Abundance (%)	Frequency (%)	N	Abundance (%)	Frequency (%)
**Native**							
*Astelia waialeale*	2.5	0	0	0	29	2.9	10.6
*B*. *arguta*	0.5	552	66.9	39.4	385	37.9	13.6
*C*. *platyphyllum*	4.5	35	4.2	33.3	33	3.3	21.2
*C*. *trigynum*	5.5	29	3.5	21.2	292	28.8	66.7
*C*. *fauriei*	1	0	0	0	19	1.9	1.5
*C*. *kauensis*	6.5	5	0.6	9.1	2	0.2	1.5
*Coprosma waimeae*	*** ***NA	0	0	0	1	0.1	1.5
*D*. *sandwicensis*	3	5	0.06	12.1	0	0	0
*Ilex anomala*	2.5	0	0	0	8	0.8	3
*Rubus hawaiensis*	3.5	0	0	0	22	2.2	3
*S*. *tameiameiae*	4.5	0	0	0	2	0.2	3
*Unknown*	NA	7	0.8	6.1	0	0	0
*V*. *calycinum*	1.5	144	17.5	27.3	222	21.9	21.1
**Non-Native**							
*H*. *gardnerianum*	4.5	18	2.2	18.2	0	0	0
*R*. *argutus*	3	18	2.2	12.1	0	0	0
*R*. *parviflorus*	1.5	12	1.5	6.1	0	0	0
Mean number of seed species per sample			1.42			1.19	
Mean number of seeds per sample (SD)			13.74 (53.99)			11.55 (20.60)	

^b^ Seed size refers to measurement of length and width of the seed expressed in mm. Seeds with no information on seed size are represented as “NA”.

## Discussion

The replacement of native with introduced species has well-documented direct effects, but the consequences for ecological processes are less well understood [70]. Increasingly depauperate island communities may be particularly susceptible to disrupted mutualisms. We found lower rates of seed dispersal in an area where a native frugivorous thrush appears to have been locally extirpated. Furthermore, introduced white-eyes, which are the most common non-native frugivores in this montane forest, dispersed similar species as the native thrush, but in different proportions. Over the long term, these patterns could potentially change the composition of Kauai’s plant communities.

Previous studies investigating the role of native and introduced birds in seed dispersal from the Hawaiian Islands report mixed results, some of which are consistent with our findings. Following the extinction of a native thrush, introduced birds in the mesic wet forest of Maui were reported as the primary disperser of native plants, dispersing 85% of native species [8]. However, in the dry forest of Maui, non-native birds primarily dispersed seeds of non-native plants, leading to dispersal failure of native plants [41]. Additionally, studies from Hawaii Island found that non-native species incompletely fill the ecological role of the native thrush, due to little dietary overlap among native and introduced birds [71], or higher representation of small-seeded fruits in the diet of the introduced species [9].

In our study, Puaiohi and Japanese White-eye exhibited a high degree of dietary overlap, yet the two species differed in the relative proportion and characteristics of seeds dispersed. The introduced Japanese White-eye consumed several small-seeded fruits such as *B*. *arguta* and *V*. *calycinum* more frequently than Puaiohi, similar to patterns observed on other Hawaiian Islands [9,53]. Although Japanese White-eye is clearly capable of consuming larger sized fruits (Fig 3), its frequent consumption of small-seeded fruits could be attributed to its small gape width (5-8mm), which is largely used for piercing the fruits and consuming the pulp. Both *B*. *arguta* and *V*. *calycinum* are relatively large fruits (length-12mm), but are filled with many small (<1mm) seeds. Moreover, Japanese White-eye is omnivorous and its consumption of fruit may depend on the availability of other food resources such as arthropods and nectar [54,71]. In contrast, Puaiohi may be more likely to consume a variety of small to larger seeded species because of its larger body and gape size, primarily frugivorous diet, and much longer evolutionary history in Hawaii. A recent study on the captive Alala (*Corvus hawaiiensis*), a large bodied frugivore on Hawaii Island, affirms the important role of larger-bodied native birds in the dispersal and germination of 14 native fruiting species, especially those with large fruits and seeds [3].

Approximately 1/3 of the seeds consumed by Japanese White-eye diet were non-native introduced species, whereas these seeds did not appear in Puaiohi fecal samples (Table 4). These introduced plant species are all considered noxious invaders in the Hawaiian Islands [72,73], and these species have been consistently reported in Japanese White-eye diet samples from other Hawaiian islands [9,74]. The large home range size of Japanese White-eye relative to Hawaii’s thrush species [53] could further facilitate introduction and spread of these non-native invasive plant species within Kauai’s rainforest.

We documented a marked difference in the number of seeds dispersed in montane forest where Puaiohi are now extremely rare or absent compared with an area where this species is still extant. Because we do not have data on seed dispersal at Kawaikoi when Puaiohi were relatively abundant, we cannot confidently attribute these low seed dispersal rates to the loss of Puaiohi. Although fruit availability and rates of rat seed predation were similar, it is possible that differences in precipitation, elevation, or other characteristics of each site could also influence seed rain. Nonetheless, the suggestion that Puaiohi are at least partially responsible for site level differences in seed rain is supported by the greater seed rain we observed at stream trap locations at Mohihi because Puaiohi selectively use streams and adjacent habitat for nesting and foraging. As such, our findings remain suggestive that large bodied frugivores may be important not just for dispersing larger seeded species, but also a greater abundance of seeds away from the parent plant, which could have important fitness consequences. Omao (*Myadestes obscurus*), a large bodied frugivore extant on Hawaii Island, was also associated with the dispersal of greater numbers of seeds relative to introduced species [9,53]. These emergent patterns in seed dispersal in the absence of native frugivores—an overall decline in seeds dispersed, disproportionate consumption and spread of small-seeded species, and increased dispersal non-native plants–all support the prediction that the replacement of native with introduced species could alter plant community composition in Hawaii’s forests.

There are several important limitations to this study that warrant consideration in interpreting our results. First, the short duration of the fruit density and seed rain portion of the study (single season, single year) limited our ability to evaluate the effect of intra- and inter-annual variability in the role of native and introduced birds in seed dispersal. Many plants in Hawaii have extended fruiting periods, and some species may have peaked outside of the bounds of our study period. Second, it was beyond the scope of our study to measure seed fate at other important stages of dispersal (e.g. seed deposition, seedling germination and establishment). Assessing rates of recruitment is an important measure of seed dispersal effectiveness and should be a priority for future studies. In Hawaii, such studies are not trivial as they require excluding non-native mammals that otherwise predate on seeds and seedlings.

Understanding the importance of predation and herbivory by rats and pigs, respectively, relative to bird-mediated dispersal is another important knowledge gap. More than twice the number of seeds dispersed by birds were damaged by rats in this study, suggesting that rat predation may play a critical role in seed dispersal limitation. Rats are well-documented to both disperse small-seeded species and predate seeds and seedlings elsewhere in Hawaii [75], as well as consuming bird eggs and nestlings [38,42,76]. We do not have sufficient information to suggest whether local extinction of avian frugivores or rat predation is more disruptive to seed dispersal on Kauai. Yet, certainly even if rats were eradicated, bird dispersal is critical to moving seeds away from parent plants [12]. Because recovering Puaiohi is a conservation priority in its own right, rat removal could help achieve this goal and benefit plant communities by reducing both competition and predation.

Finally, fecal samples were opportunistically collected across diverse years and months. Because of a relatively small sample size, we were unable to evaluate potential inter or intra-annual changes in the diet of Japanese White-eye and Puaiohi. It was also beyond the scope of our study to target less common introduced frugivorous birds (e.g., White-rumped Shama, Northern Cardinal and Melodious Laughing-Thrush), from which we were able to collect very few samples. To our knowledge, there are few previous studies documenting seed dispersal by these three species in Hawaii. Northern Cardinals consumed several fruits of native trees in dry forest [40], and seeds were found in stomach contents of five Melodious Laughing-Thrush [8], but the authors of these studies both declared the role of these birds as seed dispersers to be limited relative to the ubiquitous Japanese White-eye. We urge investigators capturing forest birds on Kauai and on others Hawaiian Islands to systematically collect fecal samples (a noninvasive procedure) to improve understanding of the ecological role of both common and lesser known introduced species.

This study provides insight into the potentially important ecological role of the critically endangered Puaiohi. In the absence of any functional equivalent, the local or global loss of similar frugivorous birds has already resulted in dispersal failure in other locations [41,77]. We document a decline in seed dispersal in areas without Puaiohi, and suggest that a common introduced bird is an incomplete ecological replacement for an endemic species, as they dispersed different distributions of seeds. These findings suggest an uncertain trajectory for Hawaii’s diverse native plant communities. Our study provides further impetus to conserve and restore Puaiohi populations both to sustain this rare endemic species and to better understand and preserve the processes that maintain Kauai’s montane forests.

## Supporting information

S1 Table Relative abundance of plant species and fruit in two study sites on Kauai. Statistically significant differences among relative plant and fleshy-fruit abundance are indicated with an asterisk (*).
(DOCX)

S2 Table Dietary overlap between native (Puaiohi) and introduced (Japanese White-eye) frugivorous birds. Overlap was calculated using Pianka’s index.
(DOCX)
