# Supplementary material for: Potential disruption of seed dispersal in the absence of a native Kauai thrush
Source: PLoS One. 2018 Jan 30;13(1):e0191992. doi: 10.1371/journal.pone.0191992 (PMC5790251; doi:10.1371/journal.pone.0191992)
Supplement: S1 Table — Statistically significant differences among relative plant and fleshy-fruit abundance are indicated with an asterisk (*). (DOCX) [file pone.0191992.s001.docx]

**S1 Table. Relative abundance of plant species and fruit in two study sites on Kauai. Statistically significant differences among relative plant and fleshy-fruit abundance are indicated with an asterisk (*****) sign.**

| Plant Species | Relative abundance (%) of plant species | | Relative abundance (%) of fleshy-fruit | |
| --- | --- | --- | --- | --- |
|  | Kawaikoi | Mohihi | Kawaikoi | Mohihi |
| *Broussaisia arguta* | 1.93 | 8.82 | 0.00 | 0.30* |
| *Cheirodendron platyphyllum* | 2.45 | 10.22 | 15.90 | 51.03 |
| *Cheirodendron trigynum* | 4.12 | 17.23 | 76.09 | 42.18 |
| *Clermontia faureri* | 1.05 | 0.00 | 0.01 | 0.00 |
| *Clermontia sp.* | 0.88 | 0.20 | 0.03 | 0.00 |
| *Coprosma waimeae* | 0.00 | 0.40 | 0.00 | 0.00 |
| *Cyrtandra longifolia* | 1.05 | 1.60 | 0.00 | 0.00 |
| *Dianella sandwicensis* | 4.65 | 8.22 | 0.00 | 0.00 |
| *Dubautia raillardioides* | 1.14 | 0.80 | 0.00 | 0.00 |
| *Elaeocarpus bifidus* | 0.44 | 0.80 | 0.38 | 0.00 |
| *Hedychium gardnerianum* | 67.48 | 5.41* | 1.71 | 0.00* |
| *Ilex anomala* | 0.09 | 0.40 | 0.00 | 0.00 |
| *Kadua terminalis* | 1.05 | 0.60 | 0.06 | 0.00 |
| *Melicope anisata* | 0.26 | 1.60 | 0.00 | 0.00 |
| *Melicope clusiifolia* | 3.68 | 9.42 | 3.99 | 5.60 |
| *Myrsine lessertiana* | 0.09 | 1.00 | 0.00 | 0.08 |
| *Psidium cattleianum* | 0.88 | 0.00 | 0.48 | 0.00 |
| *Rubus argutus* | 0.09 | 0.80 | 0.00 | 0.00 |
| *Rubus hawaiensis* | 0.00 | 2.00 | 0.00 | 0.00 |
| *Scaevola glabra* | 1.05 | 0.40 | 0.74 | 0.02 |
| *Smilax melastomifolia* | 1.58 | 1.80 | 0.21 | 0.02 |
| *Stenogyne purpurea* | 0.00 | 0.20 | 0.00 | 0.01 |
| *Styphelia tameiameiae* | 0.35 | 5.21* | 0.00 | 0.00 |
| *Syzigium sandwicensis* | 1.23 | 5.01 | 0.32 | 0.71 |
| *Vaccinium calycinum* | 4.47 | 17.64 | 0.07 | 0.05 |
| *Vaccinium dentatum* | 0.00 | 0.20 | 0.00 | 0.00 |
