# Supplementary material for: Potential disruption of seed dispersal in the absence of a native Kauai thrush
Source: PLoS One. 2018 Jan 30;13(1):e0191992. doi: 10.1371/journal.pone.0191992 (PMC5790251; doi:10.1371/journal.pone.0191992)
Supplement: S2 Table — Overlap was calculated using Pianka’s index. (DOCX) [file pone.0191992.s002.docx]

**S2 Table: Dietary overlap between Puaiohi and Japanese White-eye. Overlap calculated using Pianka’s index.**

| **Niche Overlap** |  |  |  |
| --- | --- | --- | --- |
| Pianka’s index (observed) |  |  | 0.83 |
| **Simulated indices** |  |  |  |
| Mean of simulated index |  |  | 0.14 |
| Variance of simulated index |  |  | 0.38 |
| P (observed ≥ simulated) |  |  | 0.002* |
